# A Consensus-Driven Algorithm for AECOPD Management in Croatian Emergency Departments: Preliminary Initiative

**DOI:** 10.3390/jcm15218325

**Published:** 2026-10-26

**Authors:** Anamarija Štajduhar, Andrea Vukic Dugac, Marijana Janković Danolić, Emilija Lozo Vukovac, Ivan Gornik

**Affiliations:** 1 Clinic for Respiratory Diseases, University Hospital Centre Zagreb, 10000 Zagreb, Croatia; astajduh@kbc-zagreb.hr; 2 School of Medicine, University of Zagreb, 10000 Zagreb, Croatia; igornik@kbc-zagreb.hr; 3 Medical Affairs, AstraZeneca Croatia, 10000 Zagreb, Croatia; marijana.jankovicdanolic@astrazeneca.com; 4 Clinic for Respiratory Diseases, University Hospital Centre Split, 21000 Split, Croatia; emilija.lozo@gmail.com; 5 School of Medicine, University of Split, 21000 Split, Croatia; 6 Emergency Department, University Hospital Centre Zagreb, 10000 Zagreb, Croatia

**Keywords:** acute exacerbation of chronic obstructive pulmonary disease, emergency department, clinical algorithm, treatment escalation, discharge planning, Croatia

## Abstract

**Background/Objectives:** Acute exacerbations of chronic obstructive pulmonary disease (AECOPD) are a frequent cause of emergency room (ER) visits, yet assessment, treatment, and discharge processes may lack standardization. This initiative aimed to identify perceived practice gaps and develop a consensus-based algorithm for AECOPD management in Croatian ERs. **Methods:** In 2025, 20 ER physicians from University Hospital Centres Zagreb and Split completed a structured questionnaire, addressing challenges in AECOPD recognition, assessment, treatment, and discharge. Findings were considered alongside Croatian and international COPD guidelines during three iterative, structured multidisciplinary workgroup meetings. The algorithm was tailored to local clinical practice and healthcare organization, with a predefined consensus threshold of more than 80% agreement. **Results:** The main perceived gaps concerned pharmacological treatment optimization and escalation (90%), discharge planning and follow-up (85%), and decisions regarding non-invasive ventilation, treatment escalation, and hospital admission (80%). These findings served as a basis for developing the clinical algorithm covering early recognition, differential diagnosis, severity assessment, severity-based management, maintenance treatment optimization, and discharge and follow-up recommendations. The algorithm was disseminated through printed posters in 35 major Croatian hospitals, professional society websites, and presentations at national congresses. **Conclusions:** This initiative translated identified practice gaps, evidence-based recommendations, and multidisciplinary expert input into a pragmatic algorithm for AECOPD management in Croatian ERs. It provides a proposed framework to support standardized assessment, treatment, discharge planning, and referral. Given the small, non-random sample, the findings should be interpreted cautiously. Further evaluation is needed to assess implementation, uptake, and potential effects on clinical practice and patient outcomes.

## 1. Introduction

Chronic obstructive pulmonary disease (COPD) is a condition resulting from complex interactions between genetic and environmental factors that cause airway inflammation, oxidative stress, protease–antiprotease imbalance, and progressive lung damage, leading to persistent airflow limitation, and respiratory symptoms that significantly impact patients’ quality of life and reduce life expectancy [1,2]. Tobacco smoking is the leading risk factor for COPD and a contributor to cardiovascular (CV) diseases, further complicating COPD management and patient outcomes through the close pathophysiological interplay between pulmonary and CV diseases [3,4].

Currently, COPD remains a major health concern and is the third leading cause of death worldwide, especially in countries with a lower socioeconomic status [5]. In Croatia, it poses a substantial public health burden, affecting approximately 7% of the general population and 10% of those over 40 years old, while being an underdiagnosed disease [6]. Recent national estimates rank COPD as the eighth leading cause of death, with approximately 1600 annual deaths [6]. High smoking rates in nearly 38% of the adult population and over 40% of the 25–34 age group, as well as COPD diagnoses at advanced stages when 40–50% of lung function has already been lost, limit opportunities for meaningful clinical improvement and further contribute to disease burden in Croatia [6].

COPD also imposes a considerable economic and social burden worldwide [7,8]. In the European Union, it accounts for approximately 56% of total costs related to respiratory diseases, equivalent to €38.6 million annually [9,10]; further increases in costs are projected in the coming years [7,8,10]. Importantly, acute exacerbations of COPD (AECOPDs) are the primary cause of these expenses [10].

AECOPDs are one of the most common pulmonary emergencies leading to emergency room (ER) visits [11], and approximately 50% of cases require hospitalization [12]. Although international guidelines provide recommendations for exacerbation assessment and treatment, management of AECOPD in emergency departments remains less well characterized than inpatient care, with significant variability in clinical evaluation, hospitalization decisions, discharge planning, and treatment recommendations [12]. This variability may contribute to inconsistent patient outcomes, suboptimal use of healthcare resources, and inadequate continuity of care after discharge.

Physicians managing patients with AECOPD in Croatian ERs face significant challenges in implementing guidelines, stratifying patients by disease severity, and planning discharge. A recent national study found that suboptimal adherence to Global Initiative for Chronic Obstructive Lung Disease (GOLD) recommendations and marked heterogeneity in clinical practice, highlighting the need for a more standardized approach [13].

To address this gap, we launched a quality improvement and service development initiative to standardize the management of AECOPD in ER settings across Croatia. The project aimed to identify barriers to the routine management of patients with AECOPD in the ER through a survey of physicians involved in acute care, and to develop a consensus-based clinical algorithm and discharge pathway to support patient evaluation, treatment, and follow-up. The proposed algorithm is intended for use by ER physicians, internists, and family medicine physicians involved in AECOPD management, particularly those in training or working in settings where rapid, standardized decision-making is essential.

## 2. Materials and Methods

### 2.1. Study Design, Settings, and Stakeholders

The preliminary study was conducted at two tertiary care centers in Croatia: University Hospital Centre (UHC) Zagreb, and UHC Split, in 2025. The initiative to develop an algorithm for AECOPD management in ER settings was guided by previously published Croatian data that identified gaps in the emergency department management and patient discharge for AECOPD, particularly inconsistencies in discharge recommendations and adherence to guideline-based care [13]. The algorithm development process consisted of two steps: (1) identification of unmet needs in current management of AECOPD and discharge processes through a mixed-methods approach (structured questionnaire and qualitative interviews with open-ended questions), and (2) definition of clinical gaps and barriers based on the survey outcomes and algorithm development.

Firstly, two pulmonologists experienced in the acute care of AECOPD developed a qualitative and quantitative survey to identify gaps and barriers in the routine management of AECOPD in the ER setting. The questions were shared with physicians working in the ER via an interview to gather qualitative findings and a questionnaire for quantitative results. Secondly, a multidisciplinary steering group comprising five specialists in pulmonology, intensive care, immunology and emergency medicine was gathered to reach a consensus on the identified clinical gaps and to develop the algorithm for AECOPD management in the emergency departments through three iterative cycles. The final version of the algorithm was distributed to ERs in hospitals across Croatia.

### 2.2. Survey for Unmet Needs Assessment

In the first semester of 2025, 20 physicians working in the integrated ER admission unit of the UHC Zagreb and UHC Split were invited and agreed to participate in the preliminary study. All participants were selected by convenience sampling; specifically, the investigators approached physicians working in the emergency departments and asked whether they were willing to participate in the study. The participants were specialists in emergency medicine, pulmonology, and internal medicine, as well as physicians-in-training. The survey included questions with a 5-point Likert agreement scale (1 = strongly disagree; 2 = disagree; 3 = neither agree nor disagree; 4 = agree; and 5 = strongly agree), as well as open-ended questions (Appendix A). Main topics covered recognition, diagnosis, and management of AECOPD, including pharmacological treatment (drug selection, dosing, and duration), indications for antibiotic therapy, treatment escalation, supportive care, discharge pharmacotherapy, hospitalization criteria, and post-discharge management. Participants were also invited to describe challenges encountered in routine clinical practice and to suggest potential improvements in AECOPD management.

Data were collected using a paper questionnaire. Participants completed the questionnaire on-site in the presence of an investigator, who was available to clarify any questions and led the qualitative interviews. Participation was voluntary, and no personal identifying information was collected to maintain anonymity. Consent to participate was implied by completing the questionnaire and responding to investigator’s questions, after receiving information about the study. This approach adheres to ethical principles governing the protection of participants’ personal data.

Following completion of data collection, one of the investigators performed the statistical analysis of all completed questionnaires. The purpose of the questionnaire was to explore the views of ER physicians who encounter patients with AECOPD in their clinical practice and to gain insight into their perceptions related to the management of these patients, rather than to objectively assess their clinical competence.

Responses were summarized using the median with interquartile range (IQR) as the primary measure of central tendency for ordinal Likert-scale data; the mean ± standard deviation (SD) was also reported to facilitate interpretation. Responses to the open-ended questions were independently reviewed by members of the steering group and discussed during the consensus meetings to identify common themes and practice variations. Closed-ended questions were analyzed using frequencies and percentages, with the denominator determined by the ntumber of valid responses to each item. Responses to the open-ended questions were summarized descriptively to reflect participants’ reported experiences and perceived practice gaps. This process constituted a descriptive synthesis rather than a formal qualitative analysis. Only frequencies directly supported by the available responses were retained.

### 2.3. Consensus Process and Algorithm Development

After tabulating the findings from the questionnaire, three rounds of structured workgroup meetings were conducted to reach consensus on the identified practice gaps and to develop a clinical algorithm for the management of AECOPD in ER. The consensus process was based on iterative discussion of the available evidence, current clinical guidelines, questionnaire findings, and the clinical experience of the participating experts without formal Delphi process. A predefined threshold of >80% agreement among workgroup members was established for consensus.

During the first meeting, each co-author presented their clinical experience and the main challenges encountered in the management of patients with AECOPD in the ER setting. The findings from the physician questionnaire were subsequently reviewed and discussed by the workgroup. Based on these findings and the available evidence, a core set of clinical domains and key recommendations to be addressed by the algorithm was proposed. During the development of the algorithm, one of the co-authors was responsible for selecting the relevant literature. The literature considered was narrowed down to the GOLD 2025 and 2026 Reports for the sections on symptoms of AECOPD, differential diagnosis, symptom assessment, and indications for the intensive care unit and treatment [5,14]; the Croatian national COPD guidelines for the treatment section [1]; the European Respiratory Society (ERS)/American Thoracic Society (ATS) guideline on COPD exacerbations for the treatment section [15]; the Spanish COPD Guidelines (GesEPOC) for the treatment section [16]; the AECOPD Discharge Letters document for the section on recommendations at discharge from the emergency department [13]; and the Rome Proposal for COPD exacerbations for the section on symptoms of AECOPD [17]. The algorithm incorporated a 0–10 Visual Analogue Scale (VAS) for dyspnoea assessment, consistent with the Rome Proposal, with higher scores indicating greater dyspnoea severity. The recommendations were further adapted to local routine practice in the ER setting and the Croatian hospital system, based on the expert team’s experience and clinical judgment, particularly regarding patient follow-up and existing pathways for referral to a pulmonologist depending on local administrative specificities.

During the second meeting, a preliminary version of the algorithm was presented to the workgroup. Each co-author reviewed the proposed recommendations and provided comments and suggestions regarding their clinical relevance, applicability, and clarity. Recommendations were discussed until agreement was reached, with >80% agreement considered indicative of consensus. The algorithm was subsequently revised based on the agreed-upon modifications and graphically formatted in collaboration with a professional graphic designer.

At the third meeting, the final version of the algorithm was presented, followed by a discussion of future objectives, potential research initiatives, and strategies for the dissemination and implementation of the algorithm. Each recommendation and the overall structure of the algorithm were discussed, and final agreement was confirmed according to the predefined consensus threshold of >80%.

In addition to the three main meetings, which were conducted in a hybrid format, both on-site and online, continuous communication among the authors was maintained throughout the intervening period to facilitate an efficient and timely development process.

The algorithm was designed to standardize physician-led management in the emergency departments and was tailored to local resource availability and the organization of care. Interventions intended to be performed independently by patients or caregivers were excluded from the algorithm.

### 2.4. Artificial Intelligence Usage

Artificial intelligence (AI)-based tools were used only in the preliminary stages of the study to assist with translation, and grammatical editing. All authors actively participated in the conceptualization and development of the manuscript and carefully reviewed and edited the initial content generated or assisted by AI-based tools. The authors take full responsibility for the accuracy, integrity, and final content of the manuscript.

## 3. Results

### 3.1. Outcomes of Practice Gap Assessment

Twenty physicians completed the questionnaire, of whom 6 (30%) specialized in emergency medicine, 7 (35%) in internal medicine, 5 (25%) were residents, and 2 (10%) specialized in other specialties. The study included 11 participants from University Hospital Centre Zagreb and 9 participants from University Hospital Centre Split. The level of ER experience varied across participants: 6 (30%) reported <2 years of experience, 3 (15%) reported 2–5 years, 8 (40%) reported 5–10 years, and 3 (15%) reported >10 years. Eighteen participants (90%) provided responses to all questions. All 20 participants provided responses to the closed-ended questions. Two participants (10%) did not respond to the final two open-ended questions, resulting in 18 available responses for each question. These missing responses were excluded from the analysis of the respective questions, and no imputation was performed. Denominators were adjusted according to the number of available responses for each question.

The practice gap assessment questionnaire demonstrated neutral-to-moderate agreement across most items. Q8 “I feel confident when discharging a patient for outpatient treatment” received the highest level of agreement (mean 3.55 ± 0.89), whereas Q9 “I am familiar with escalation of chronic COPD therapy” and Q10 “I know which medications to prescribe for a newly diagnosed patient” received the lowest levels of agreement (mean 2.35 ± 0.59 and 2.20 ± 0.62, respectively). Responses to other questions were predominantly neutral, with a tendency toward agreement (Table 1). A high proportion of respondents (85%) never escalated chronic COPD therapy, and 90% reported difficulties in referring patients to a pulmonologist (Table 2).

Qualitative analysis identified several key challenges in the ER management of AECOPD:

- Inconsistent classification of exacerbation severity (15 [75%] of respondents);
- Variability in objective patient assessment, particularly regarding the use and interpretation of oxygen saturation and arterial blood gas analysis (14 [70%]);
- Unclear thresholds for noninvasive ventilation (NIV), treatment escalation, and hospital admission (16 [80%]);
- Limited optimization and escalation of pharmacological therapy in the ER (18 [90%]);
- Discharge without standardized treatment recommendations, optimization of maintenance therapy, or appropriate follow-up referral (17 [85%]);
- Communication gaps between emergency physicians, pulmonologists, and primary care physicians result in fragmented continuity of care and delayed or absent specialist follow-up (13 [65%]).

These findings identified the key areas requiring standardization and informed the subsequent consensus-building process.

### 3.2. Clinical Algorithm for AECOPD Management in ER Setting

**Figure 1:**
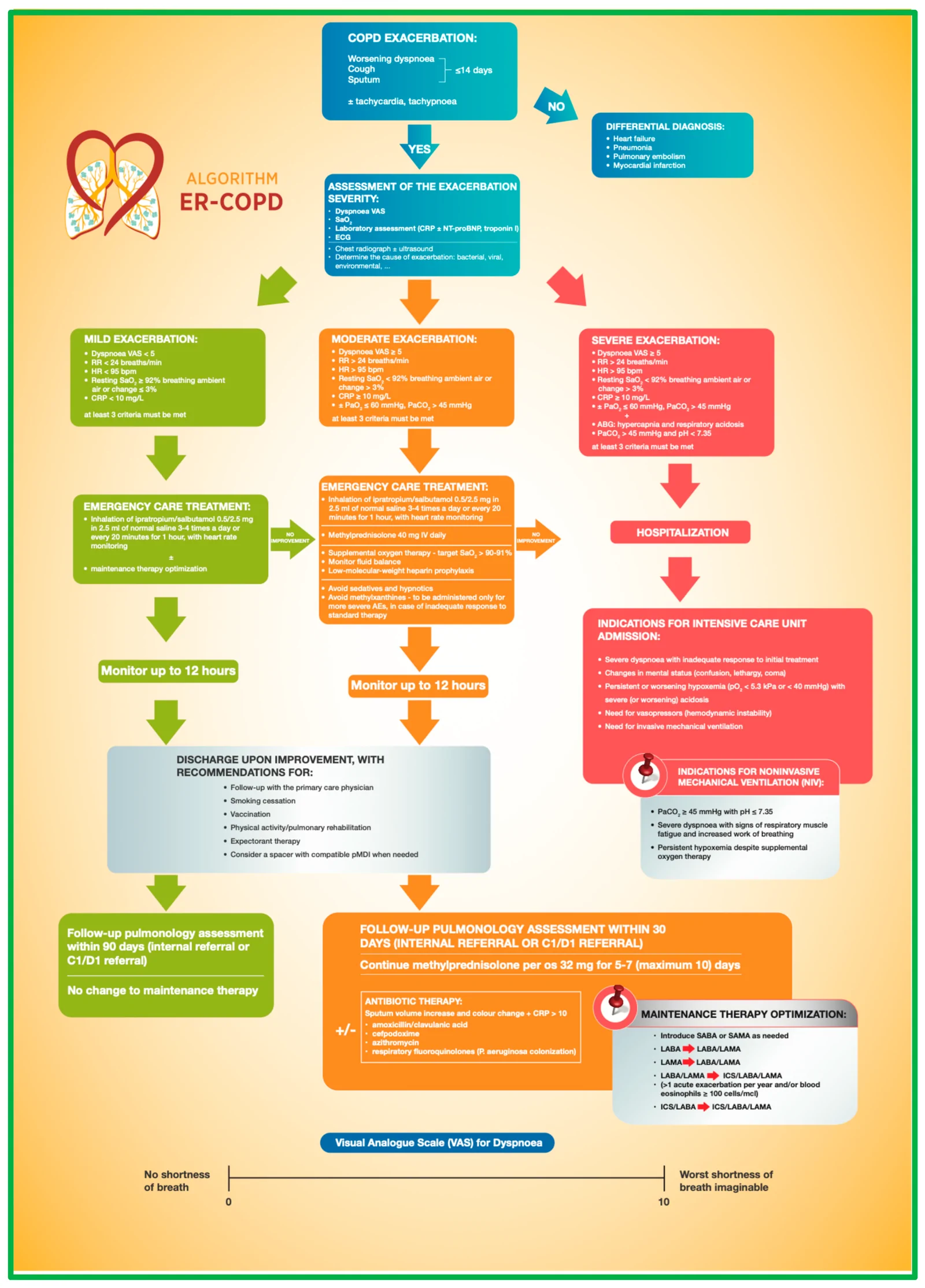
Clinical algorithm for AECOPD management in the ER setting (developed by authors in line with GOLD 2025 Global strategy for prevention, diagnosis, and management of COPD [14]). Notes: 1. Dyspnoea severity is assessed using a 0–10 Visual Analogue Scale (VAS), in accordance with the Rome Proposal [17]; 2. C1/D1 referrals are issued by primary care physicians to enable access to specific diagnostic evaluations and specialist hospital care. Abbreviations: AEs, acute exacerbations; ABG, arterial blood gas; COPD, chronic obstructive pulmonary disease; CRP, C-reactive protein; ECG, electrocardiogram; ER, emergency room; HR, heart rate; ICS, inhaled corticosteroid; IV, intravenous; kPa, kilopascal; LABA, long-acting β_2_-agonist; LAMA, long-acting muscarinic antagonist; NT-proBNP, N-terminal pro-B-type natriuretic peptide; PaCO_2_, arterial partial pressure of carbon dioxide; pO_2_, partial pressure of oxygen; RR, respiratory rate; SABA, short-acting β_2_-agonist; SAMA, short-acting muscarinic antagonist; SaO_2_, arterial oxygen saturation; VAS, visual analogue scale.

Based on key areas requiring standardization identified through the practice gap assessment and discussed during the first working group meeting (Table 1, Table 2, and qualitative findings presented above), the steering group outlined and then developed the clinical algorithm and discharge pathway (Figure 1).

The ER-COPD algorithm includes the following key consensus recommendations:

- **Early recognition of AECOPD:** Patients presenting to the ER with symptoms suggestive of AECOPD, such as worsening dyspnea, cough, or sputum for up to 14 days, with or without tachycardia and tachypnea, should undergo prompt clinical assessment based on typical symptoms and signs to facilitate early diagnosis and timely treatment initiation.
- **Differential diagnosis:** Clinicians should systematically consider alternative diagnoses and concomitant conditions, particularly CV diseases, which may precipitate AECOPD and significantly affect patient outcomes.
- **Assessment of exacerbation severity:** Patients should be classified according to exacerbation severity (mild, moderate, or severe) using standardized clinical criteria (Figure 1).
- **Initial work-up for severity assessment:** A standardized assessment should include evaluation of dyspnea severity, vital signs, peripheral oxygen saturation, and, where clinically indicated, arterial blood gas analysis, electrocardiography, laboratory investigations, chest imaging, and microbiological testing.
- **Severity-based management for AECOPD:** Patients should be managed by exacerbation severity:
  ○ ***Escalation of care for severe cases:*** Refer patients for hospitalization and assess if intensive care is needed. Indications for initiation of non-invasive mechanical ventilation should be evaluated (details included in Figure 1).
    *Note:* Non-invasive and invasive ventilation strategies are not described in detail, as these extend beyond the scope of the present algorithm and may be considered part of the intensive care unit, depending on the institution’s practice.
  ○ ***ER management for mild cases:*** This includes inhaled bronchodilator therapy with or without maintenance therapy optimization, and clinical monitoring for up to 12 h (Figure 1);
  ○ ***ER management for moderate cases:*** This includes inhaled bronchodilator therapy with heart rate monitoring, systemic corticosteroids, oxygen supplementation (when indicated), with clinical monitoring for up to 12 h (Figure 1).
- **Discharge recommendations and documentation for mild or moderate cases:** Standardized documentation should include clear recommendations regarding maintenance treatment, medication changes, secondary prevention, and specialist follow-up:
  ○ ***Systemic corticosteroids:*** Prescribe according to current recommendations regarding indication, dose, and duration of therapy (Figure 1).
  ○ ***Antibiotic therapy:*** Prescribe at discharge only when clinically indicated for patients with *moderate* AECOPD (Figure 1).
  ○ ***Optimization of maintenance therapy:*** Review and adjust before discharge for patients with *moderate* AECOPD to reduce the risk of future exacerbations (Figure 1).
  ○ ***Secondary prevention:*** Provide individualized recommendations regarding pneumococcal vaccination, smoking cessation, risk factor modification, pulmonary rehabilitation (where appropriate), and symptomatic therapy to facilitate sputum clearance, and use of a spacer device as needed.
  ○ ***Follow-up after discharge:*** Plan specialist follow-up before discharge, with referral pathways aligned with the Croatian healthcare system to ensure continuity of care:
    - *Mild cases:* Pulmonology assessment within 90 days
    - *Moderate cases:* Pulmonology assessment within 30 days.

Overall, the management of AECOPD should be personalized, multidimensional, and, within ER settings, delivered as rapidly and efficiently as possible while maintaining patient safety. In more complex cases, a multidisciplinary approach is required, involving collaboration among emergency physicians, intensive care, pulmonologists, cardiologists, and infectious disease specialists.

**Figure 2:**
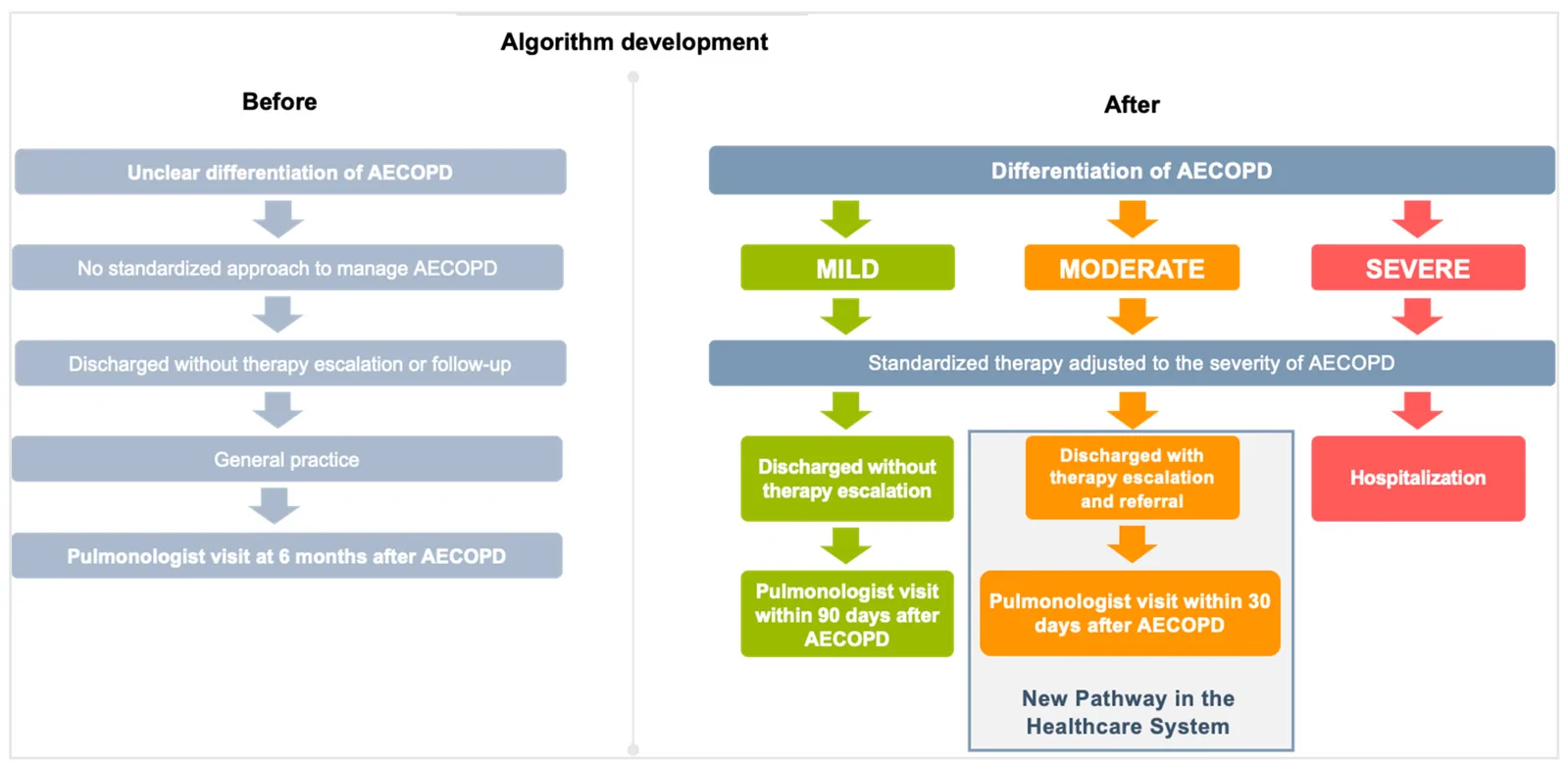
Anticipated patient journey before and after implementation of the clinical algorithm for AECOPD management. Abbreviations: AECOPD, acute exacerbations of chronic obstructive pulmonary disease.

A summary of the patient journey before and after algorithm development is shown in Figure 2.

The algorithm was successfully disseminated in a poster format to emergency departments of the 35 largest hospitals in Croatia. The algorithm was also presented at national congresses of internal medicine and pulmonology and is publicly available in a printed format.

## 4. Discussion

This quality improvement initiative identified important sources of heterogeneity in the management of AECOPD across ER physicians and translated these findings into a standardized clinical algorithm and discharge pathway tailored to Croatian routine clinical practice.

The practice gap assessment identified inconsistencies in the assessment of exacerbation severity, objective patient evaluation, treatment escalation, discharge planning, and communication among emergency physicians, pulmonologists, and primary care physicians. Although respondents reported moderate confidence in recognizing and assessing AECOPD, confidence in patient management was lower (mean 2.55), familiarity with escalating maintenance therapy was limited (mean 2.35), as only 15% had ever escalated COPD treatment, and 90% reported difficulties in referring patients to a pulmonologist (with an additional 5% mentioning “sometimes”). Another study conducted in Croatia in 8 hospitals found that discharge letters reported COPD severity in only 13% of cases, severity of the current exacerbation in 7%, and lacked maintenance-treatment recommendations in 18%. Although early follow-up was recommended in the majority (72%) of cases, it was formally scheduled in only one-third (32%), highlighting notable gaps in communication and continuity of care [13]. Similar gaps have also been reported internationally. A scoping review of 44 studies found generally low or mixed adherence to guidelines and inadequate discharge referral practices [18]. In an inpatient cohort in California, only half (55%) of patients received complete guideline-based therapy within 24 h, and only one-quarter (23%) received an outpatient pulmonary referral [19]. In a multicenter study in emergency departments from Israel with 344 patients hospitalized with AECOPD, lower proportions of patients received routine ER treatments (i.e., 72% of patients received short-acting bronchodilators and 55% systemic corticosteroids) [12]. These findings together with results of other studies focused on the coordinated COPD clinical management and support at multiple levels underscore the need for standardized pathways for assessment, treatment escalation, discharge planning, and continuity of care [20,21,22,23].

Additionally, appropriate treatment for AECOPD is frequently delayed due to late-seeking care of patients [24], but also due to the complexity and difficulty of AECOPD diagnosis [25]. AECOPD is a heterogeneous clinical syndrome mainly diagnosed based on subjective, nonspecific clinical features that overlap with or are confounded by concomitant pneumonia, congestive heart failure, and other acute cardiopulmonary conditions [25]. In the absence of standardized clinical pathways, management decisions in the emergency setting may also be influenced by clinician knowledge, diagnostic framing, memory, attention, and individual clinical behavior [26]. This is particularly relevant in Croatia, where physicians working in integrated ER units are not necessarily specialists in emergency medicine or pulmonology. Combined with demanding work conditions and variations in clinical experience, these factors may contribute to inconsistencies in patient management.

The importance of a standardized approach is also reinforced by the natural history of COPD. Prolonged exacerbation recovery has been associated with poorer health status, a shorter interval to the next exacerbation, and accelerated disease progression. In the London COPD Cohort, exacerbations without recovery of lung function occurred in less than 10% of cases and were associated with an additional decline in forced expiratory volume in one second (FEV_1_) of 10.8 mL/year [27]. A recent meta-analysis reported all-cause readmission rates of 18% within 30 days and 31% within 90 days following hospitalization for AECOPD [28]. Moreover, the rate of rehospitalization is alarmingly high, placing COPD alongside pneumonia and heart failure as a major contributor to healthcare utilization and a significant determinant of mortality in this patient population [28]. The longer an exacerbation persists, the greater the risk of subsequent recurrence [27]. Early recognition, prompt initiation of guideline-directed therapy, and effective discharge management in the ER are crucial.

Although comprehensive international guidelines, including the GOLD Report [14], ERS/ATS recommendations [15], and GesEPOC [16], provide evidence-based recommendations for AECOPD management, they are broad reference frameworks rather than locally adapted point-of-care tools for ER practice. The algorithm developed in this project translates current national and international recommendations into a concise, pragmatic, and user-friendly tool tailored to ER workflows. Although developed to address gaps identified in the Croatian healthcare system, it may also serve as a framework for implementation in settings facing similar challenges or can be further adapted to other local healthcare structures and clinical practice. Its primary purpose was to facilitate early patient stabilization by supporting rapid clinical assessment, diagnosis/classification in mild, moderate, and severe AECOPD, and timely initiation of evidence-based treatment. To achieve this, the algorithm integrates key clinical information obtained during the initial patient assessment, including symptoms and signs of clinical deterioration, vital signs, oxygenation status, and selected laboratory biomarkers (e.g., complete blood count, C-reactive protein, and procalcitonin) into a structured framework that supports patient assessment, risk stratification, and treatment guidance.

Beyond acute AECOPD episode management, a holistic, multidisciplinary approach is provided throughout hospitalization and after discharge. Effective management should include optimization of pharmacological therapy, standardized discharge planning, structured post-discharge follow-up, patient education, smoking cessation counseling, vaccination, and clear communication among healthcare providers. By integrating acute management with a structured discharge pathway, the proposed algorithm seeks to improve continuity of care and support more consistent implementation of guideline-based management in routine clinical practice.

It is also important to recognize that the post-exacerbation period represents a critical window of increased CV risk in patients with COPD, particularly for acute coronary syndrome, decompensated heart failure, and arrhythmias [29,30,31]. This risk is highest during the first days and weeks following an exacerbation, but may remain elevated for several months and, in some cases, up to one year after the event [29,30]. Structured post-exacerbation follow-up, together with optimization of COPD management and timely treatment of identified CV comorbidities, is therefore essential to address the overall cardiopulmonary risk and potentially improve long-term outcomes [29,31]. Recognizing the complexity of patient management with COPD, our proposed algorithm also aims to raise clinicians’ awareness of the need for ongoing follow-up and, where feasible, to facilitate earlier access to specialist pulmonology care. Accordingly, we incorporated recommendations for referral to pulmonology follow-up as an integral component of the proposed algorithm.

In Croatia, the algorithm was disseminated nationwide via printed posters, and educational lectures at national congresses of internal medicine and pulmonology in collaboration with Croatian professional societies, to facilitate its integration into routine clinical practice. Importantly, it was implemented as part of a broader national initiative to improve COPD care in Croatia, alongside the development of national guidelines, standardization of discharge documentation, and educational activities for primary care physicians. This coordinated approach may strengthen continuity of care across healthcare settings; however, dissemination alone does not ensure adoption, and future evaluation should assess uptake, adherence, and effects on clinical practice and patient outcomes.

One of the key strengths of this initiative is the translation of national and international recommendations into a concise, pragmatic, readily accessible tool for routine ER practice in Croatia. Additionally, multiple dissemination pathways, including posters, educational webinars with high participation, provide broad access to a shared evidence-based framework for AECOPD management.

The algorithm combines management of AECOPD, guideline-based therapy, and a standardized discharge process with follow-up, thereby ensuring continuity of care beyond the ER setting. Its multidisciplinary development by specialists in emergency medicine, pulmonology, intensive care, and internal medicine reflects the clinical complexity of AECOPD in patient management and the importance of concomitant respiratory, CV, infectious diseases along with external, organizational factors. Designed for routine use within the Croatian healthcare system, the algorithm provides a practical framework for more consistent clinical decision-making and communication.

This initiative has several limitations. Given the sampling methodology for the questionnaire, it is possible that physicians with less experience or confidence may have been more likely to participate, and that recruitment of physicians with greater knowledge and clinical experience could have resulted in different responses. This potential selection bias should therefore be acknowledged when interpreting the findings. Nevertheless, the questionnaire was intended only as an add-on component of the study. Its primary role was to provide an initial indication of perceived gaps and needs in the management of patients with AECOPD and to support the rationale for developing a practical algorithm as an aid to clinicians involved in the care of these patients.

The algorithm was developed specifically for the Croatian healthcare system and may require local adaptation before use in other geographies. Although the multidisciplinary working group included experts from the largest national pulmonology centers and was coordinated by the University Hospital Center Zagreb, which hosts the Croatian Ministry of Health Reference Center for Asthma and Chronic Obstructive Pulmonary Disease, its small size may have limited the breadth of perspectives represented. However, this limitation was partially mitigated by incorporating survey findings in gap assessment and by the clinical expertise available during algorithm development.

The algorithm has not yet been prospectively validated, and independent clinical content review was not performed at this stage; its effects on guideline adherence, revisits at emergency departments, hospital readmissions, and other patient outcomes remain unknown. Finally, to maintain simplicity and usability, it focuses on key diagnostic and therapeutic decisions and does not address complex clinical scenarios, including non-invasive ventilation. Variable selection was primarily informed by the Rome Proposal [17] and the current Croatian national COPD guidelines [1]. Future updates may be needed following implementation outcome studies.

## 5. Conclusions

Managing acute exacerbations of COPD remains difficult and often involves doctors from different specialties, many of whom do not regularly care for these patients. Differences in clinical experience, communication challenges, and poor continuity of care can lead to inconsistent management and less-than-ideal patient outcomes. To address these issues, we created a practical, evidence-based algorithm that converts current national and international guidelines into a clear and easy-to-use pathway for emergency department management of AECOPD.

Besides aiding acute clinical decisions, the algorithm encourages consistent treatment plans, standardized discharge procedures, and better continuity of care. By offering a concise and accessible tool designed for the Croatian healthcare system, it aims to help implement guidelines, reduce unnecessary practice variations, and enhance care quality and consistency.

The next step involves testing the algorithm in routine clinical practice. Future studies should evaluate its effect on guideline adherence, ER decision-making, hospital admissions and readmissions, exacerbation frequency, systemic corticosteroid use, discharge safety, mortality, healthcare utilization, and costs. These studies are crucial to determine if this approach leads to an effective implementation strategy that results in measurable improvements.

## Figures and Tables

**Table 1:** Summary of survey results on self-reported familiarity and confidence in managing AECOPD in the ER setting (N = 20).

Question	Median (IQR)	Mean ± SD
Q3: I am able to reliably recognize a COPD exacerbation	3 (3–4)	3.45 ± 0.69
Q4: I am able to assess the severity of a COPD exacerbation	3 (3–4)	3.30 ± 0.92
Q5: I feel confident in managing patients with COPD exacerbation.	2 (2–3)	2.55 ± 0.94
Q6: I am able to select appropriate medications for COPD exacerbation	3 (3–3)	3.35 ± 0.67
Q7: I am familiar with appropriate dosing and duration of therapy	3 (3–4)	3.40 ± 0.94
Q8: I feel confident when discharging a patient for outpatient treatment	3 (3–4)	3.55 ± 0.89
Q9: I am familiar with escalation of chronic COPD therapy	2 (2–3)	2.35 ± 0.59
Q10: I know which medications to prescribe for a newly diagnosed patient	2 (2–3)	2.20 ± 0.62

Abbreviations: COPD, chronic obstructive pulmonary disease; N, total number of participants; IQR, interquartile range; SD, standard deviation.

**Table 2:** Distribution of responses to open-ended questions from the survey (N = 20).

Question	n (%)
Q11: Have you ever escalated chronic therapy for COPD?	
Yes	3 (15%)
No	17 (85%)
Q12: Do you encounter difficulties when referring patients to a pulmonologist?	
Yes	18 (90%)
Sometimes	1 (5%)
No	1 (5%)

Abbreviations: COPD, chronic obstructive pulmonary disease; N, total number of participants; IQR, interquartile range; SD, standard deviation.

## Data Availability

The data presented in this study are available upon request from the corresponding author.

## Abbreviations

The following abbreviations are used in this manuscript:

ABG	Arterial blood gas
AE	Acute exacerbation
AECOPD	Acute exacerbation of chronic obstructive pulmonary disease
COPD	Chronic obstructive pulmonary disease
CRP	C-reactive protein
CV	Cardiovascular
ECG	Electrocardiogram
ER	Emergency room
ERS/ATS	European Respiratory Society/American Thoracic Society
FEV_1_	Forced expiratory volume in 1 s
GesEPOC	The Spanish COPD Guidelines
GOLD	Global Initiative for Chronic Obstructive Lung Disease
HR	Heart rate
ICS	Inhaled corticosteroid
IQR	Interquartile range
IV	Intravenous
kPA	kilopascal
LABA	Long-acting β_2_-agonist
LAMA	Long-acting muscarinic antagonist
NIV	Noninvasive ventilation
NT-proBNP	N-terminal pro-B-type natriuretic peptide
PaCO_2_	Arterial partial pressure of carbon dioxide
pO_2_	Partial pressure of oxygen
RR	Respiratory rate
SABA	Short-acting β_2_-agonist
SAMA	Short-acting muscarinic antagonist
SaO_2_	Arterial oxygen saturation
SD	Standard deviation
VAS	VAS, visual analogue scale

## Appendix A

**Table A1:** Structured Interview & Questionnaire for Emergency Room Physicians.

Assessment of Clinical Decision-Making in COPD Exacerbations	
Instructions: Please answer the following questions based on your clinical experience in the emergency department.	
**Section 1. Assessment of Clinical Decision-Making in COPD Exacerbations**	
1. Medical specialty:	
	Emergency Medicine Internal Medicine Family Medicine Resident Other:
2. Years of experience in the emergency department:	
	<2 2–5 5–10 >10
**Section 2. Knowledge and Clinical Competence (Likert scale: 1–5)** 3. I am able to reliably recognize a COPD exacerbation. [1 2 3 4 5] 4. I am able to assess the severity of a COPD exacerbation. [1 2 3 4 5] 5. I feel confident in managing patients with COPD exacerbation. [1 2 3 4 5] 6. I am able to select appropriate medications for COPD exacerbation. [1 2 3 4 5] 7. I am familiar with appropriate dosing and duration of therapy. [1 2 3 4 5] 8. I feel confident when discharging a patient for outpatient treatment. [1 2 3 4 5] 9. I am familiar with escalation of chronic COPD therapy. [1 2 3 4 5] 10. I know which medications to prescribe for a newly diagnosed patient. [1 2 3 4 5]	
**Section 3. Clinical Practice** 11. Have you ever escalated chronic therapy for COPD?	
	Yes No
12. Do you encounter difficulties when referring patients to a pulmonologist?	
	Yes No Sometimes
**Section 4. Open-ended Questions** 13. What challenges or uncertainties do you encounter in managing COPD exacerbations? ----------------------------------------------- 14. Please list any additional questions or dilemmas? -----------------------------------------------	
